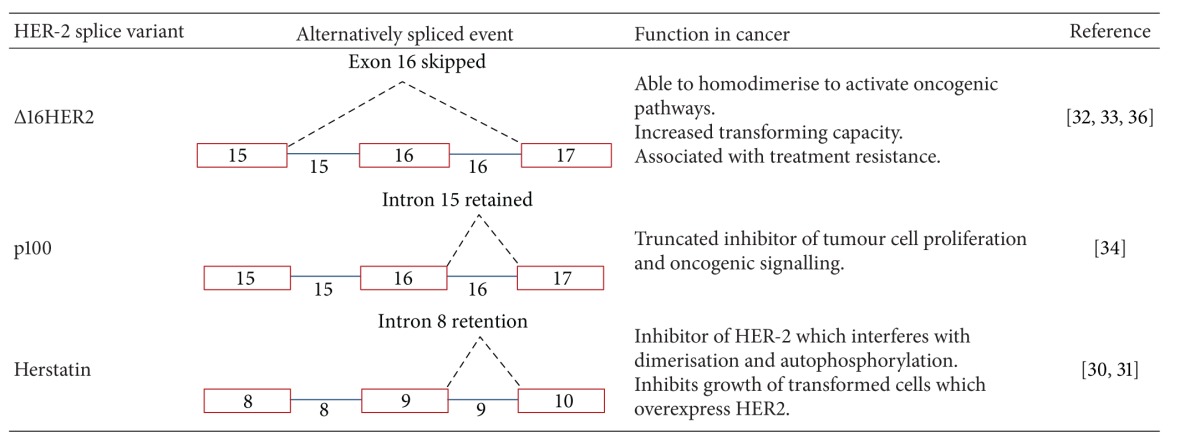# Clinical Significance of HER-2 Splice Variants in Breast Cancer Progression and Drug Resistance

**DOI:** 10.1155/2013/973584

**Published:** 2013-07-01

**Authors:** Claire Jackson, David Browell, Hannah Gautrey, Alison Tyson-Capper

**Affiliations:** ^1^Institute of Cellular Medicine, Faculty of Medical Sciences, Newcastle University, Newcastle upon Tyne NE2 4HH, UK; ^2^Queen Elizabeth Hospital, Gateshead, Tyne and Wear NE9 6SX, UK

## Abstract

Overexpression of human epidermal growth factor receptor (HER-2) occurs in 20–30% of breast cancers and confers survival and proliferative advantages on the tumour cells making HER-2 an ideal therapeutic target for drugs like Herceptin. Continued delineation of tumour biology has identified splice variants of HER-2, with contrasting roles in tumour cell biology. For example, the splice variant Δ16HER-2 (results from exon 16 skipping) increases transformation of cancer cells and is associated with treatment resistance; conversely, Herstatin (results from intron 8 retention) and p100 (results from intron 15 retention) inhibit tumour cell proliferation. This review focuses on the potential clinical implications of the expression and coexistence of HER-2 splice variants in cancer cells in relation to breast cancer progression and drug resistance. “Individualised” strategies currently guide breast cancer management; in accordance, HER-2 splice variants may prove valuable as future prognostic and predictive factors, as well as potential therapeutic targets.

## 1. Introduction

**Figure 1:**
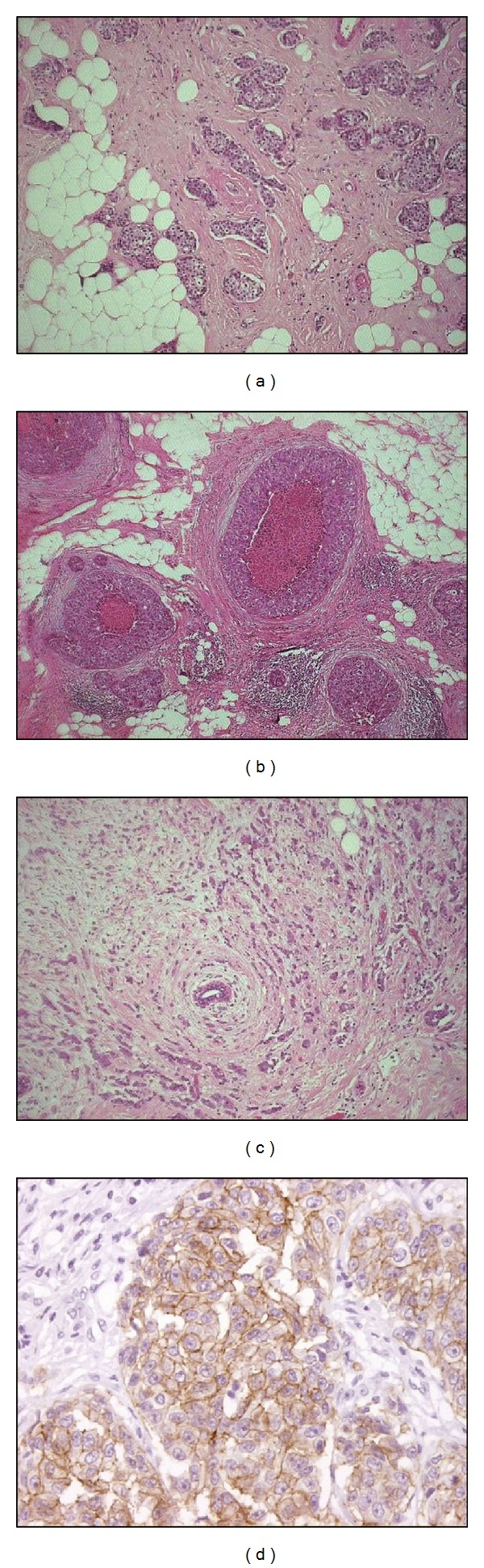
Histological images of breast carcinoma. Images of (a) ductal carcinoma no special type (NST), (b) ductal carcinoma in situ (DCIS), and (c) lobular carcinoma. (d) HercepTest positive staining: immunocytochemical staining indicates HER-2 overexpression in invasive breast cancer (Images courtesy of Dr. D. Hemming, Queen Elizabeth Hospital, Gateshead).

Breast cancer is a heterogeneous disease comprising subtypes of varied morphology, prognostic profiles, and clinical outcomes [1, 2]. Tumours arise from malignant transformation of hyperplasic epithelia within the breast [3], and numerous mutagenic changes contribute to the transformation process which abnormally alters the cellular environment. Atypical hyperplasic cells may progress to carcinoma in situ, categorised as ductal carcinoma in situ (DCIS) or lobular carcinoma in situ (LCIS) [3] (Figure 1). These terms denote malignant cells restricted to ducts or acini of lobules. Carcinoma becomes invasive when atypical cells penetrate the basement membrane and spread into the surrounding stroma [3] (Figure 1). Cancer cells then have the potential to spread to surrounding skin or muscles or to metastasise to axillary lymph nodes or distant sites such as bone, liver, and brain where new tumours may form [3]. 

In recent decades, there has been a paradigm shift from increasingly extensive and invasive surgery to “cure” and prevent relapse to conservation surgery with lower morbidity and the use of adjuvant therapy to eliminate “micrometastases.” This approach improved survival, reduced the risk of recurrence, and minimised the impact of treatment on quality of life thus emphasising a need for more directed treatment strategies [4].

Consequently, there has been a subsequent shift in more recent years to “individualized” treatment with better therapeutic targeting. The advent of the humanised monoclonal antibody trastuzumab (commonly referred to as Herceptin) which targets human epidermal growth factor-2 (HER-2) transformed management of breast cancer patients [5]. Patients whose tumours are shown to overexpress HER-2 now undergo more rigorous treatment, with Herceptin and chemotherapy. This modernised approach of “targeted” treatment now guides cancer management with attempts to tailor therapeutics to specific tumours [4]. 

## 2. HER-2: Structure and Function

**Figure 2:**
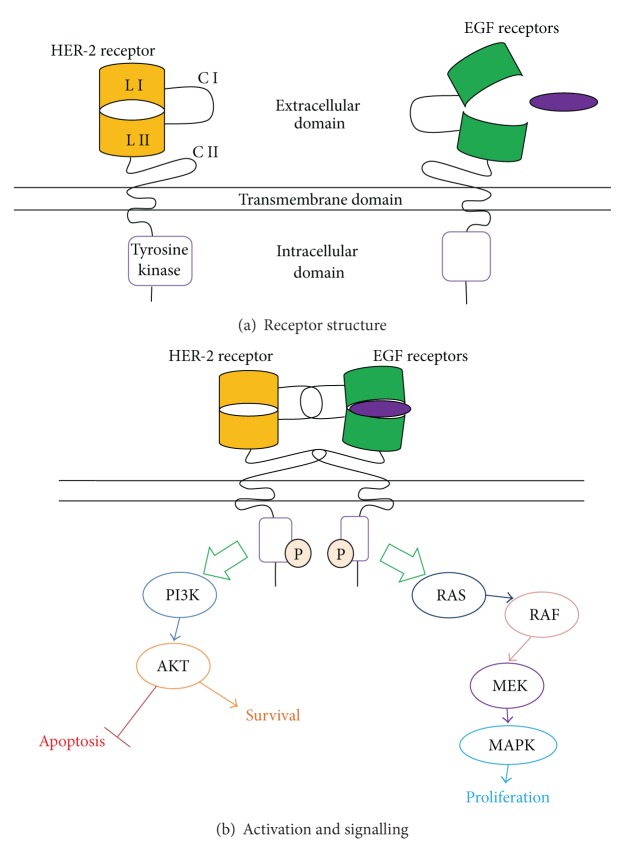
Schematic of HER-2 structure, activation, and signalling. (a) HER-2 is a single transmembrane cell surface receptor with extracellular, transmembrane, and intracellular regions. The extracellular region comprises of two ligand-binding domains (L I and L II) and two cysteine-rich domains (C I and C II) [8]. Intracellularly, HER-2 receptors have intrinsic tyrosine kinase activity (TK). (b) HER-2 does not bind ligands but is activated by forming heterodimers with other ErbB receptors via interaction at the cysteine-rich domains. This results in autophosphorylation of the tyrosine kinase domains and induction of downstream signalling. Normal signalling includes stimulation of the PI3K/AKT pathway which induces survival mechanisms and inhibits apoptosis, whilst the RAS/RAF/MEK/MAPK pathway stimulates cell proliferation [8].

HER-2 is a 185 kDa transmembrane cell surface receptor of the human epidermal growth factor (EGF) family [6]. There are four receptor members of this family: HER-1 (EGFR, ErbB-1), HER-2 (ErbB-2), HER-3 (ErbB-3), and HER-4 (ErbB-4). EGF receptors have a highly conserved extracellular domain, a transmembrane domain, and an intracellular domain with tyrosine kinase activity [7] (Figure 2). Ligand-receptor binding induces conformational changes and receptor dimerisation via interaction at both extracellular cysteine-rich regions [7, 8]. This results in autophosphorylation and kinase activation [8]. EGF receptor signalling has important roles in cell proliferation, differentiation, and survival [9] (Figure 2). 

Thirteen ligands interact with EGF receptors. HER-1 and HER-4 may actively homodimerise. HER-2 and HER-3 are nonautonomous as HER-2 has no known ligand, and HER-3 lacks tyrosine kinase activity [8]. HER-2 and HER-3 therefore form heterodimers with other EGF receptors to promote signal induction (Figure 2). 

HER-2 was first identified in 1984 by Schechter et al. [6] and has since been recognised as the “preferred” dimerisation partner [10]. Whilst it lacks the “typical” ligand-binding structure, HER-2 sustains an active conformation acting as a potent coreceptor for other EGF receptors [8]. Prolonged dimer interaction consequently sustains downstream survival and proliferative signalling [10]. 

## 3. HER-2: Insights into Tumour Biology 

Each subtype of invasive breast cancer is associated with certain clinical characteristics and treatment options. Although the umbrella term of “breast cancer” remains, the discovery of new biomarkers and gene expression profiling prompted a move to consider subtypes of breast cancer as different diseases within their own right [11]. HER-2-positive breast cancer is typically more aggressive with a poorer prognostic outlook [12]. HER-2 is routinely measured in clinical practice, and patients whose tumours score 3+ on HercepTest immunocytochemical staining (Figure 1(d)) and test positive for amplification of the *HER-2* gene using fluorescence in situ hybridization (FISH) will be offered treatment with Herceptin in combination with chemotherapy. 

**Figure 3:**
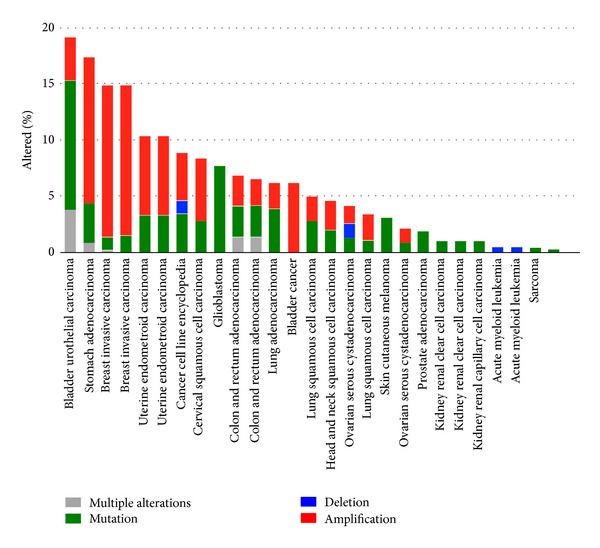
Patterns of HER-2 in different cancers. Bar chart shows genetic changes in a wide range of different tumours and cancer types, including mutation, deletion, and amplification. Note that amplification is particularly prevalent in invasive carcinoma of the breast. Data was generated using the cBIO Cancer Genomics Portal [14, 15].

*HER-2* has been acknowledged as a protooncogene since a mutated form, the *NEU *oncogene, was isolated using cell transformation studies in the rat that used tumour DNA [13]. Moreover, amplification of the *HER-2 *gene occurs in a number of different cancers and is particularly prevalent in invasive carcinoma of the breast (Figure 3) [14–16]. HER-2 protein is overexpressed in many human cancers and associated with 20–30% of breast cancers [7, 17]. High levels of the receptor result in enhancement of oncogenic signalling pathways [12]. Consequently, HER-2-positive tumours are associated with increased metastatic potential, poor prognosis, and recurrence [18, 19].

As HER-2 is expressed at much higher levels in certain tumours (Figure 3) than in normal tissue and plays a key role in mitogenic and antiapoptotic signalling [7, 12, 20], it was recognised as an ideal target for anticancer drugs. Current approved therapies include the aforementioned monoclonal antibody Herceptin and tyrosine kinase inhibitor lapatinib. Such agents fostered improved survival rates with five-year survival now at 84% for women in England [21]. However, whilst these drugs improve breast cancer treatment, they are still not fully understood and a continuing challenge [5, 11, 12]. For example, it is still unclear why some patients do not respond to Herceptin as a single agent, and also why initial responders regress within 6 months [12, 22]. There are also some HER-2-positive patients who relapse early, and their more common pattern of metastatic disease involves spread to the bone, liver, and lungs, whilst there are also long-term responders who can relapse with the less commonly seen metastases to the brain. Further exploration of HER-2 biology, signaling, and resistance mechanisms is therefore essential to develop and implement new strategies of therapeutic intervention.

## 4. HER-2 Splice Variants and Cancer Biology

Many cancer-related changes in alternative splicing have been identified to distinguish splicing patterns in “normal” breast compared to cancer samples [23–26]. Cancer-specific events can result in proteins with “procancer” properties which may promote malignant transformation or confer a survival advantage on cancer cells, such as resistance to treatment [27–29]. In recent years, focus has been directed at the level of the transcriptome with one area of continued investigation centred on the different variants of HER-2 that can be produced by alternative splicing [30–33].

To date, three naturally occurring HER-2 spliced variants in breast cancer have been reported (Table 1), namely, Δ16HER-2, Herstatin, and p100. As new therapeutic strategies are devised in efforts to tackle current problems with treatment resistance, attention has been directed to further unravel the impact of HER-2 with particular focus on these spliced variants [30]. Studies have investigated the transforming, oncogenic and drug-resistant activities of these isoforms [30, 33–35].

## 5. Δ16HER-2 

Δ16HER-2 arises from the in-frame deletion of exon 16; a 48 bp cassette exon which encodes a small region of the extracellular domain of HER-2 [32]. Resultant loss of cysteine residues in the extracellular domain of HER-2 induces a conformational change, promoting homodimerisation via intermolecular disulfide bonds [32, 33, 36]. Castiglioni et al. propose a causal role of Δ16HER-2 in cancer development suggesting that malignant transformation occurs once the proportion of Δ16HER-2 expressed reaches a specific threshold [32]. Conversely, wild-type HER-2, whilst relevant, is not considered sufficient to induce transformation [32, 37]. In addition, numerous studies have linked Δ16HER-2 with resistance to trastuzumab advocating the use of tyrosine kinase inhibitors as an alternative [32, 33].

Δ16HER-2 appears to constitute a more aggressive variant compared to wild-type HER-2. Not only has it been purported to be important in malignant transformation, but research also suggests a role in disease progression. Mitra et al. reported that 89% of patients with HER-2-positive breast tumours, in whom disease progresses to local lymph nodes, expressed Δ16HER2 [33]. This suggests that patients expressing Δ16HER-2 may benefit from more aggressive therapeutic intervention.

## 6. P100

Scott et al. first described an HER-2 mRNA variant encoding a protein constituting only the extracellular domain of the full-length protein [38]. Termed p100, this splice variant interferes with the oncogenic activity of wild-type HER-2 and arises via an in-frame stop codon as a result of intron 15 retention [34]. Studied in cell lines and tumours derived from breast cancer and gastric cancer, p100 has the capacity to inhibit tumour cell proliferation [34, 38]. Further exploration reported a decrease in downstream signal induction such as the MAP kinase pathways [30]. 

Several studies have provided evidence that this secreted truncated form of HER-2 may serve as a serum biomarker particularly in informing treatment decisions [30, 39, 40]. Leyland-Jones et al. demonstrated reduced levels of p100 expression in more aggressive tumours [39]. Further studies in breast cancer have continued to evaluate its role as a biomarker, and its value remains an issue for debate [30, 41]. Some reports suggest that this variant may compete for selection by monoclonal antibodies such as trastuzumab thereby interfering with its treatment activity [38, 41].

## 7. Herstatin

Herstatin is another naturally occurring truncated HER-2 protein generated from alternative HER-2 mRNA transcripts that retain intron 8 [42]. This secreted HER-2 variant, like p100, contains only the extracellular domain of the full-length protein and has a novel C-terminus of 79 amino acids [42]. Several lines of evidence demonstrate that Herstatin can act as an inhibitor of full-length HER-2, since it is able to interfere with dimerization, decrease tyrosine phosphorylation, and consequently inhibit the growth of transformed cells which overexpress HER-2 [43]. Interestingly, the autoinhibitory properties of Herstatin can also impede HER-2 activity by preventing transactivation of its hetesrodimeric partner HER-3; Herstatin does this by specifically disrupting HER-2/HER-3 and also HER-2/EGFR dimer phosphorylation [31, 43]. Since Herstatin has been perceived to be a “protective” HER-2 variant rather than an “oncogenic” protein, its expression profile has been assessed in normal versus tumour tissues [35]. Findings from this study, not surprisingly, show that Herstatin levels are significantly higher in noncancerous breast cells compared to carcinoma cells.

## 8. Clinical Implications of HER-2 Splice Variants

Prior to the advent of specific markers, such as HER-2, and drugs like Herceptin, cancer management was directed by tumour grade and status alone. Treatments were not specifically targeted, for example, the use of chemotherapeutic agents which target cell division. Today, finding ways to further exploit tumour biology is central to overcoming challenges to current diagnostics and managing as well as developing new “individualized” interventions with improved stratification to treatment. 

Δ16HER-2 has also been implicated in resistance of HER-2-positive tumours to anti-HER-2 therapies [33]. Therefore, measurement of this variant may also be especially informative in predicting response to treatment with anti-HER-2 therapies.

This is somewhat intriguing considering the aggressive nature of HER-2-positive tumours, and p100 has been reported to decrease with increasingly aggressive tumours [39]. In view of this, it would be of value to compare the proportions of p100 (and Herstatin mRNA) and protein between tumour samples to accurately determine how expression varies with the “aggressiveness” of a tumour. As these HER-2 splice variants secrete proteins [34], in future studies it may be of value to obtain corresponding patient serum samples along with tumour samples to gain a more accurate representation of their protein levels. Additional variables which contribute to clinical outcome, such as hormone receptor status or lymph node involvement, would also need to be considered.

Potential presence of other truncated HER-2 proteins should also be considered when interpreting HER-2 protein expression. Truncated proteins arise not only from alternative splicing but also via proteolytic cleavage or alternative initiation of translation [44]. HER-2 proteins encoding only the extracellular domain (ECD) are produced, ranging from 95 to 105 kDa [44]; therefore, it cannot be assumed that all 100 kDa HER-2 proteins are p100 as they may constitute other HER-2 ECD-derived proteins. 

## 9. What Is a “True” HER-2 Status?

Hormone receptor status can be predictive of the efficacy of endocrine therapies, but we now know that current screening strategies, using immunochemistry and N-terminal antibodies, may overlook the “true” hormonal receptor status since the procedure does not take into account truncated splice variants of either the oestrogen receptor or progesterone receptor [26, 45, 46]. The same principle can be applied to HER-2 status. As HER-2 positive status is determined when immunocytochemical staining exceeds a specified threshold (Figure 1(d)), tumours deemed that HER-2 negative may not be wholly negative but do not exceed this “threshold of positivity.” Previous reports by Castiglioni et al. demonstrated that the proportion of Δ16HER-2 expressed was central to malignant transformation [32]. In DCIS samples where exon 16 skipping occurs, Δ16HER-2 may have been a trigger to transformation. Although HER-2 status is not routinely measured in DCIS, Harada et al. reported that HER-2 positivity in DCIS patients was associated with increased risk of developing invasive carcinoma [47]. This is especially relevant when previous reports regarding the cancer-related and treatment resistance properties of Δ16HER-2 are considered [30, 33]. The proportion of Δ16HER-2 has already been shown to be important in breast cancer progression [33, 34]. If a DCIS sample was shown to express high levels of Δ16HER-2, this patient may be at greater risk of disease progression and therefore may benefit from more rigorous treatment or followup. 

Previous studies report that p100 expression decreases in more aggressive tumours [39]; DCIS is considered a less aggressive form of breast cancer as it is preinvasive therefore unable to metastasise. Such results align with expectations that p100 expression is higher in less aggressive tumours. These spliced variants may play a role in determining the nature and clinical outcome of breast tumours in which they are expressed. 

It remains to be fully explored as to whether coexpression of the mRNA of the three HER-2 spliced variants has any impact on subsequent translation, or indeed how the proteins collectively might interact when coexpressed. It would be of value to determine the proportions of all three HER-2 splice variants in the same tumour cells and to evaluate their impact on cell growth and drug resistance. One study has evaluated full-length HER-2 status using qPCR [48] and advocated its use in concordance with immunohistochemistry; however, it did not consider quantification of HER-2 splice variants. 

## 10. HER-2 Variants as Clinical Targets?

One potential line of development for targeted anticancer therapeutics is the manipulation of HER-2 spliced variants [49, 50]. Splicing-targeted therapeutics has already shown a promise in treatment of disease. For example, induced exon skipping in Duchenne muscular dystrophy produces a “Becker muscular dystrophy-like dystrophin isoform,” successfully reducing disease severity [51]. One study has also demonstrated success using splice-switching oligonucleotides (SSO) to target HER-2 [52]. Wan et al. reported that SSO-induced skipping of exon 15 produced a novel protein, Δ15HER2, which acted to downregulate wild-type HER-2 and induce apoptosis of HER-2 overexpressing tumour cells [52]. Such strategies could be adapted to manipulate production of Δ16HER-2 or p100. Whilst research is ongoing to improve delivery methods of splicing-targeted therapies [53], they do appear as a promising strategy for future anticancer therapeutic intervention. 

Detecting the proportion and relevance of HER-2 spliced variants, as described, could potentially “redefine” HER-2 status. These spliced variants could consequently impact treatment routes in HER-2-positive tumours and also HER-2-negative tumours and DCIS. For example, tumours previously deemed HER-2 negative which express that these variants above a specified threshold may in fact benefit from therapies targeting the HER-2 spliced variant thereby improving stratification of patients to “individualized” treatments. Additionally, proportions of splice variants in patients who do not respond to, or regress on, anti-HER-2 drugs may indicate treatment with alternative drugs as a superior alternative.

This could potentially have implications regarding the value of HER-2 spliced variants in a clinical context. The presence or absence of HER-2 spliced variants may influence prognosis or response to treatment. Further investigation could reveal a clinical use for Δ16HER-2, Herstatin, or p100, for example, in making treatment decisions or as a potential therapeutic target. Further exploration of HER-2 biology, signaling, and resistance mechanisms is therefore essential to develop and implement new strategies of therapeutic intervention.

## Figures and Tables

**Table 1:** HER-2 spliced variants and their role in cancer.